# Corroles-Porphyrins: A Teamwork for Gas Sensor Arrays

**DOI:** 10.3390/s150408121

**Published:** 2015-04-08

**Authors:** Rosamaria Capuano, Giuseppe Pomarico, Roberto Paolesse, Corrado Di Natale

**Affiliations:** 1 Department of Electronic Engineering, University of Rome Tor Vergata, Via Politecnico 1, 00133 Roma, Italy; E-Mail: capuano@ing.uniroma2.it; 2 Department of Chemical Science and Technology, University of Rome Tor Vergata, Via della Ricerca Scientifica, 00133 Roma, Italy; E-Mails: pomarico@scienze.uniroma2.it (G.P.); roberto.paolesse@uniroma2.it (R.P.)

**Keywords:** corroles, porphyrins, quartz microbalance, volatile organic compounds

## Abstract

Porphyrins provide an excellent material for chemical sensors, and they have been used for sensing species both in air and solution. In the gas phase, the broad selectivity of porphyrins is largely dependant on molecular features, such as the metal ion complexed at the core of the aromatic ring and the peripheral substituents. Although these features have been largely exploited to design gas sensor arrays, so far, little attention has been devoted to modify the sensing properties of these macrocycles by variation of the molecular aromatic ring. In this paper, the gas sensing properties of a porphyrin analog, the corrole, are studied in comparison with those of the parent porphyrin. Results show that changes in the aromatic ring have important consequences on the sensitivity and selectivity of the sensors and that porphyrins and corroles can positively cooperate to enhance the performance of sensor arrays.

## 1. Introduction

Porphyrins are aromatic macrocycles constituted by four pyrrole rings linked by methine bridges. The four inner core nitrogen atoms represent probably the most versatile ligand system, able to coordinate almost all the elements of the Periodic Table. The peripheral positions of the macrocycle can be further decorated by additional peripheral substituents. The interplay between the aromatic ring, the metal ion, and the peripheral groups gives rise to specific and unique functionalities that can be exploited in a number of different applications.

Porphyrins chemistry is widely used in nature [1]. In animals, a porphyrin iron complex, the heme, is the prosthetic groups of several metalloproteins, involved in functions that are essential for life. For example the heme binds molecular oxygen in hemoglobin, stores it in myoglobin, and activates O_2_ in cytochrome P450 enzymes. On the other hand, magnesium complexes of reduced porphyrins, the chlorins, where one or two peripheral double bonds are saturated, are the key chromophores involved in the photosynthetic processes.

Such a richness of chemical and physical phenomena prompted the use of porphyrins in artificial systems. In particular the binding properties are of great interest for the development of chemical sensors. Porphyrins offer a wide range of interaction mechanisms that can be exploited to bind host molecules either airborne or in solution. The interaction mechanisms include van der Waals forces, hydrogen bond, *π*-cation, and coordination to the central metal ion. In chemical sensors, solid-state layers of porphyrins are formed at the interface between the physical transducers and the environment carrying the molecules to be detected. Porphyrins are commonly used with transducers measuring either the mass or the optical properties such as light absorption and luminescence.

Porphyrins have also a strong tendency to aggregate by *π* − *π* interaction in the solid layers, and the formation of these supramolecular architectures offer further binding mechanism with target analytes [2]. It is interesting to note that in the solid state all the above-mentioned porphyrin-analyte interactions can take place at once. This leads to the fact that porphyrins based sensors are rather unselective. On the other hand, the great advantage of porphyrins is that the interactions can be modulated by changing the molecular building blocks. This property can be exploited to design sensor arrays that requires sensors broadly-selective but with a different sensitivity pattern. The paradigm of chemical sensor arrays is the sense of olfaction and the chemical sensor arrays are widely known as electronic noses [3].

As mentioned above, the peripheral substituents, and the metal ion can be efficiently modified to optimize the sensitivity of the sensor [4]. Porphyrins have been demonstrated as excellent sensing materials for electronic nose applications aimed at medical diagnosis, food control, and environmental monitoring [5].

The properties of porphyrins can be extended to macrocyclic analogs, where the basic molecular skeleton of the porphyrin ring is modified, by changing either the number of pyrroles or the bridges between the pyrrole units. Among the different porphyrin analogs, in recent years corrole have attracted an increasing interest, due to some peculiar properties shown by this macrocycle. Corrole shares the molecular skeleton of corrin, the nucleus of Vitamin *B*_12_, with two pyrrole rings directly linked, but retaining an aromatic character like porphyrins. This results in a more rich *π*-electron system than porphyrin, which contribute to confer to corrole distinct properties of catalytic activity, sensing, and photochemistry [6].

In terms of chemical sensors properties, the modification of the molecular skeleton is a way to modulate the sensing properties of the macrocyclic receptors, mimicking what is done in nature where porphyrins activate O_2_ and chlorins are used for photosynthetic processes. Since a strict structure-properties relationship is operating for these macrocycles, the modification of the aromatic ring provides a further degree of freedom to modulate the sensing behaviour of the molecular receptor. Although this tool is expected to improve the performance of sensor arrays, corroles have only been occasionally used as gas sensors. Barbe et al. demonstrated the preferential binding of carbon monoxide on Co(III) corroles [7], and dihydroxyphenyl-substituted corroles have also demonstrated a high affinity for this gas [8]. On the other hand, Wang studied the sensing behaviour of corroles coated single wall carbon nanotubes for NO_2_ detection [9]. In other cases, corroles have complemented porphyrins in electronic noses, for instance for the diagnosis of lung cancer [10].

In spite of these studies, a deep comparison of sensors based on porphyrins and corroles has not been done. In this paper, corroles and porphyrins are compared considering two basic macrocycles: tetraphenylporphyrin and triphenylcorrole as free-base, and complexed with manganese and iron. The molecules were applied as solid films on quartz microbalance sensors and the sensing properties were evaluated by exposing the sensors to vapors of ethanol, triethylamine, ethyl acetate, and dimethylformamide. Results illustrate the different behaviour of the free bases molecules and the different relationship between metal ion and sensitivity in porphyrins and corroles.

## 2. Experimental Section

**Figure 1:**
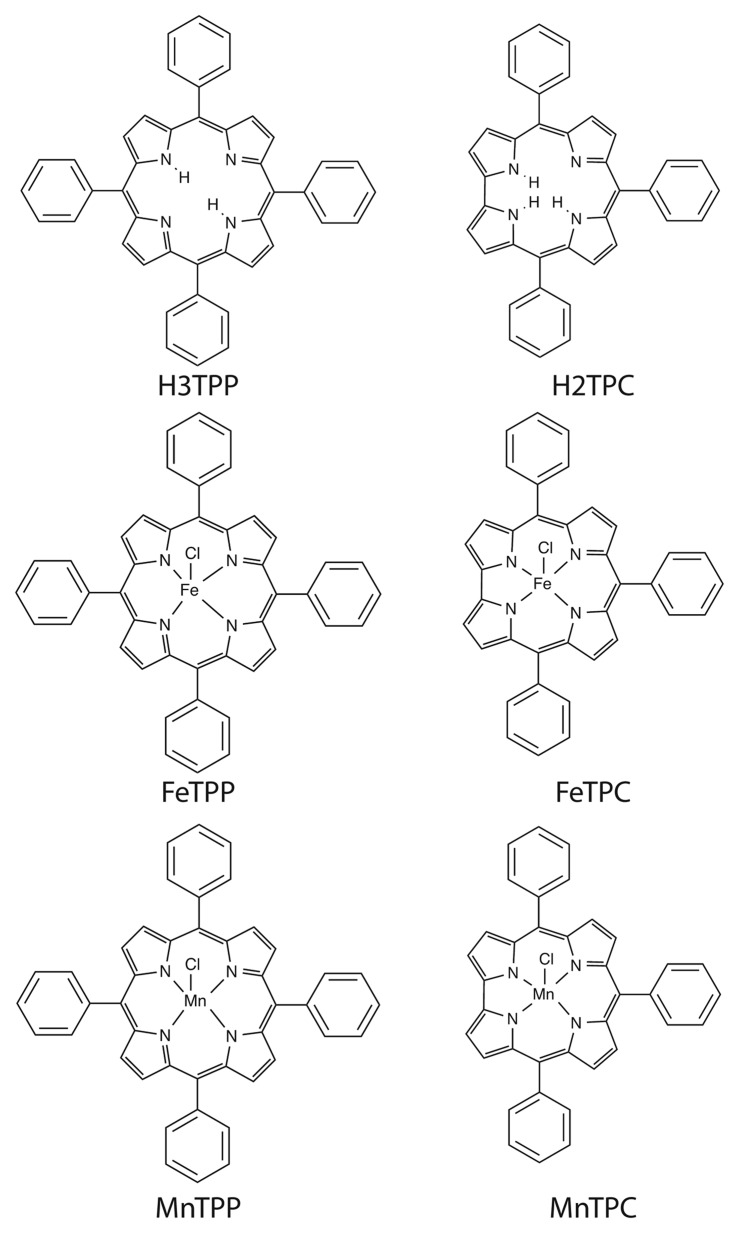
Schemes of the studied macrocycles.

Figure 1 shows the molecular structures of the investigated corroles and porphyrins. Meso-phenyl substituted derivatives have been considered for both macrocycles. For both porphyrin and corrole the free base, and iron and manganese complexes have been studied. Manganese and Iron have been chosen for their relevant catalytic activity and coordination chemistry.

5,10,15,20-tetraphenylporphyrin (H2TPP), [11] 5,10,15-triphenylcorrole (H3TPC) [12]. The corresponding, iron: [5,10,15,20-tetraphenylporphyrinato] Iron Chloride (FeTPP); 5,10,15-triphenylcorrolato] Iron Chloride (FeTPC), and Manganese: [5,10,15,20-tetraphenylporphyrinato] Manganese Chloride (MnTPP); 5,10,15-triphenylcorrolato] Manganese Chloride (MnTPC), complexes have been prepared according to literature methods [13,14].

Quartz microbalance (QMBs) were AT-cut quartzes oscillating in the thickness shear mode, with a fundamental frequency of 20 MHz; the crystal diameter is 7.0 mm and the gold electrodes diameter is 5.0 mm. QMBs sensors are mass transducers [15], where the resonance frequency shift is, in the small perturbations regime, linearly correlated to the mass loading. The used quartz has a nominal mass sensitivity of about 4.8 Hz/ng, considering a minimum reliable frequency measurement of 1 Hz, it corresponds to a mass resolution of approximately 0.2 ng [16]. Thin films of corroles and porphyrins were deposited by spray-coating technique on both the sides of the quartz disks from 10^−3^ mol/L in CHCl_3_ solutions. The mass deposited in each coating corresponded to a frequency shift of 45 KHz. This quantity offers a good compromise between the sensor response (the amount of absorbed molecules) and the QMB linearity in terms of frequency shift and energy loss. The six prepared sensors were housed in a stainless steel cell with a volume of 10 mL. Each sensor was connected to an electronic oscillator circuit and the signal frequencies were sequentially measured by an integrated frequency counter.

The sensors were tested with vapors of ethanol, triethylamine, ethyl acetate, and dimethylformamide. The liquid were kept at the constant temperature of 303 K, and the saturation pressure was diluted with a carrier of gas nitrogen at dilution percentage from 3% to 15% by mass-flow controllers always maintaining the total flux to 200 sccm.

## 3. Results and Discussion

**Figure 2:**
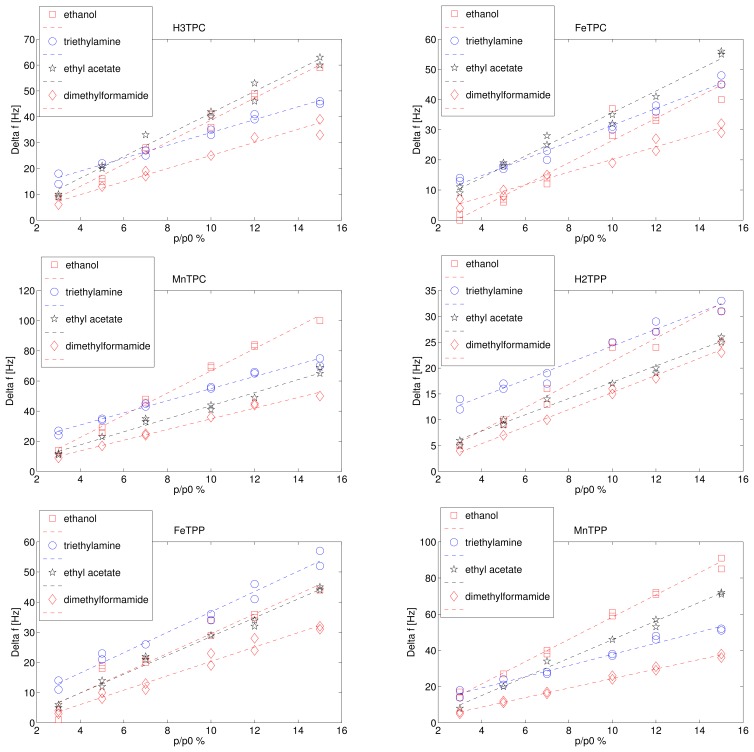
Response curves of the six sensors as a function of the relative concentration (*p*/*p*_0_) of the tested volatile compounds.

**Figure 3:**
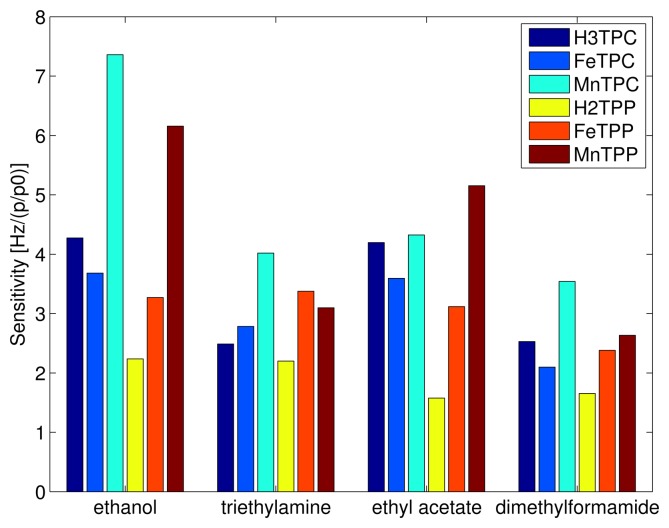
Sensitivity, in units of Hz per relative concentration, of the sensors to the four tested vapors.

Figure 2 shows the frequency shift as a function of the saturation pressure dilution for the four tested compounds. Each exposure was measured twice, and both the responses are plotted in Figure 2. Since the saturation pressure is different for each volatile compound, the volatile concentrations in the gaseous phase are different. The largest vapour pressure is achieved by ethyl acetate (11.86 KPa), the smallest by dimethylformamide (0.51 KPa) while the vapour pressure of ethanol and triethylamine are rather similar being 5.95 KPa and 6.89 KPa respectively. The vapour pressure indicates the concentration of the volatile compound in air and it is inversely proportional to the sticking coefficient of the volatile compounds in case of van der Waals forces. The ratio *p*/*p*_0_ can be called relative concentration and it is usually utilised to describe the concentration of water vapour in air (relative humidity). The sensor responses are linearly proportional to the relative pressure in the investigated range. The slope of the linear fit is the sensitivity of the sensor response with respect to the relative concentration of the volatile compounds. The sensitivities are shown in Figure 3.

The different behaviour of the macrocycles can be captured considering that the sensitivity of free base corrole (H3TPC) exceeds the sensitivity of the free base porphyrin (H2TPP). In case of ethanol and ethyl acetate the sensitivity of corrole is more than twice with respect to porphyrin while the sensitivities to the other two compounds are barely identical. The enhancement of selectivity of free base corrole with respect to free base porphyrin correlates with the molecular polar surface area (PSA).

**Figure 4:**
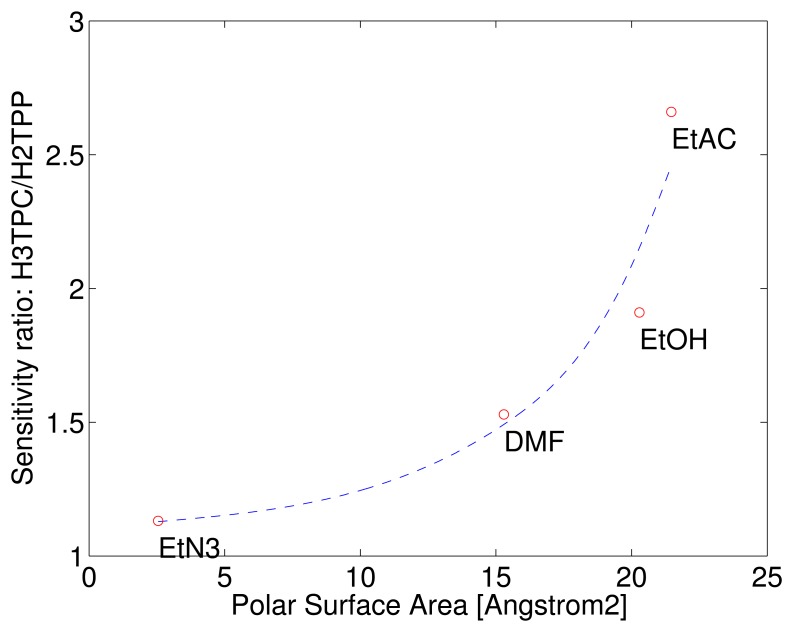
Ratio of the sensitivities of free base corrole and porphyrin as a function of the polar surface area (PSA) of the tested compounds. Each point is labeled with the corresponding compound: EtN3: triethylamine; DMF: dimethylformamide; EtOH: ethanol; EtAC: ethyl acetate.

PSA is a molecular indicator defined as the total surface contribution of the polar atoms of a molecule. PSA is used in quantitative structure-activity relationship (QSAR) models where it is used to predict the membranes penetrability of drugs [17]. PSA also correlates with the gas adsorption in molecular organic frameworks [18]. The PSA of the four tested vapours have been calculated with Spartan 14 (Wavefunction Inc., Irvine, CA, USA) using the semi-empirical PM3 method to determine the equilibrium geometry. Figure 4 shows the ratio of sensitivities as a function of the volatile compound PSA. The correlation with the polar area of the sensitivity of corrole with respect to porphyrin may be interpreted considering that the additional hydrogen in the corrole ring reduces the molecular symmetry making more probable the occurrence of polar interactions.

The two macrocycles show a different behaviour when the metal ion is complexed. In the case of porphyrins, for all the compounds the sensitivity of metal complexes is larger than the sensitivity of the free base. MnTPP exceeds the sensitivity of FeTPP in case of ethanol, ethyl acetate, and dimethylformamide. Among corroles, MnTPC is always the most sensitive. It is interesting to observe that the inclusion of iron in TPC increases the sensitivity towards the compound for which the free base corrole has the lowest sensitivity, then triethylamine is the only case where FeTPC exceeds H3TPC.

A further insight in the comparison between corroles and porphyrins is obtained considering the sensors as members of a sensor array. In this case an efficient illustration of the array properties and the contributions of each sensor to the whole array can be gained by principal component analysis (PCA) [19].

In the case here illustrated each measurement corresponds to a six-dimensional vector that is represented in a 6D vector space. PCA represents the multidimensional data projecting them onto a subspace identified by the principal components which are a basis of uncorrelated variables obtained as a linear combination of the original variables. The total variance of the data is then distributed along the different principal components, and since the sensor responses are rather correlated one each other (see Figure 2), the total variance of the data can be confined to few principal components. In particular, the first three principal components account for more than 99% of the total variance.

The main results of the PCA are the scores and the loadings. The scores are the coordinates of the data points in the basis of the principal components while the loadings are the coordinates of the unit vectors of the original basis in the basis of the principal components. Since the unity vectors of the original basis are the features of the multivariate data (the sensors in our case) the loadings describe how the sensors contribute to the principal components. Scores and loadings can be displayed together in the same plot (called biplot) where the relationship between data and sensors can be explored.

**Figure 5:**
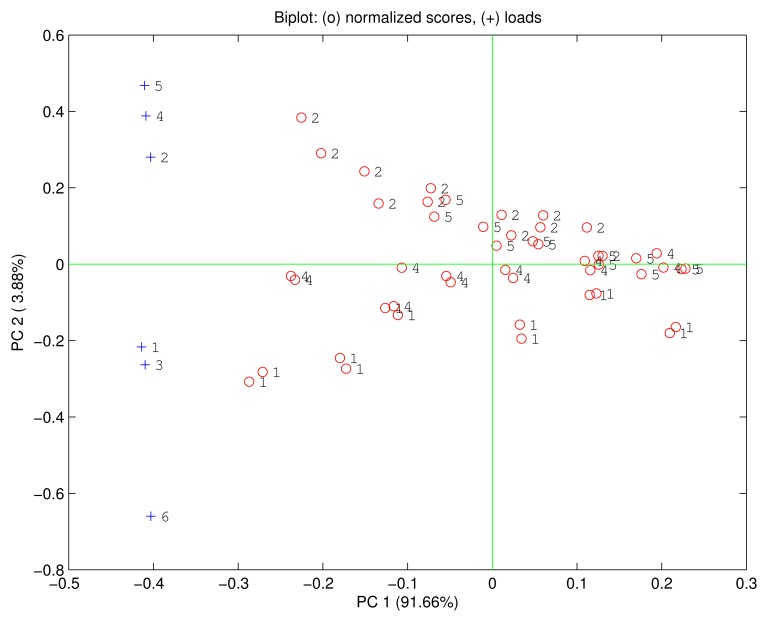
Biplot of the first two principal components. Scores identified as circles are labeled according to the compound: 1: ethanol, 2: triethylamine, 4: ethyl acetate, 5: dimethylformamide. Loadings plotted as crosses are labelled as: 1: H3TPC, 2: FeTPC, 3: MnTPC, 4: H2TPP, 5: FeTPP, 6: MnTPP. The data are ordered along the first principal component according to the concentration, the different directions identify the individual compounds.

Figure 5 shows the biplot of the first two principal components. The data points are ordered according to the relative concentration. Each compound is identified by a direction in the PC1-PC2 plane. The first principal component carries 91.66% of the total variance and it is correlated with the growing concentrations of the VOCs. Ethanol is rather isolated from the other compounds that are quite overlapped. As a consequence, in the PC1-PC2 plane only ethanol is distinct from the other compounds. The loadings of H3TPC, MnTPC and MnTPP are plotted along the ethanol direction, while the loadings of the other sensors are oriented towards the other compounds. The position of the loadings is coherent with the previous discussion about the sensors sensitivities and the dominant polar interaction in the free base corrole and the manganese macrocycles.

**Figure 6:**
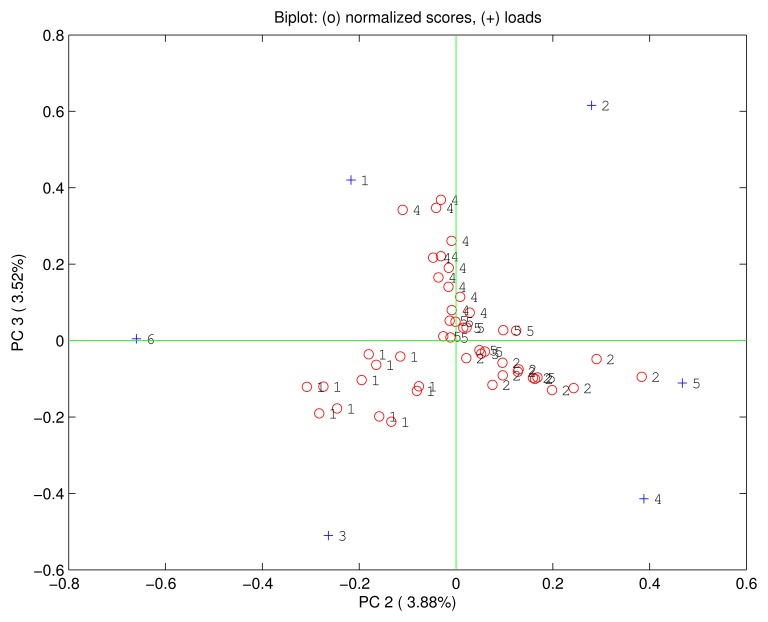
Biplot of the second and third principal components. Scores identified as circles are labeled according to the compound: 1: ethanol, 2: triethylamine, 4: ethyl acetate, 5: dimethylformamide. The loadings are marked by crosses and labelled as: 1: H3TPC, 2: FeTPC, 3: MnTPC, 4: H2TPP, 5: FeTPP, 6: MnTPP. In this plot the scores are independent on the concentration and the four compounds are clearly separated.

Being PC1 correlated with the concentration it is possible to suppose that the rest of principal components is immune from concentration effects. Figure 6 shows the biplot of the second and the third principal components where no relationship between the scores and the concentration is found. Rather in this plot, although it represent only about 7% of the total variance, the scores are clustered according to the quality of the compounds giving rise to a complete separation among the four vapours. Even the loadings are no more correlated one each other, but MnTPC and MnTPP are found oriented towards the ethanol, H2TPP and FeTPP towards the triethylamine, and H3TPC and FeTPC towards the ethyl acetate. Interestingly, the dimethylformamide is plotted at the center of the plane suggesting an equal consideration from the six sensors.

The PCA reveals the usual combination of quantity and quality in the sensors signal. Furthermore, this is a very interesting cases where quantity and quality are separated in the principal components giving rise to an effective identification of compounds independent from the concentration of the vapor.

## 4. Conclusions

Synthetic porphyrins stem from the attempt to mimic the brilliant properties of biological porphyrins, such as heme and chlorophyll. At the synthetic level the porphyrin macrocycle can be varied by coordination of different metal ions in the inner core, or by addition of functional groups in the peripheral positions. It is known that these modifications in the molecular structure alter the molecular functionalities and in particular the binding properties exploited for chemical sensing. A further possibility to modify porphyrin characteristics is to modify the macrocycle molecular skeleton. In this paper we considered the corrole, which is among the most interesting porphyrin analogs. Here, we compared the sensing properties of QMB sensors functionalized with solid layers of free base, iron and manganese complexes of tetraphenyporphyrin and triphenylcorrole. The sensors have been tested measuring different concentrations of ethanol, triethylamine, ethyl acetate, and dimethylformamide vapors diluted in molecular nitrogen. Sensors were prepared depositing the same mass of molecules on each QMB. The structural arrangement of the molecular films has not been studied in this paper where we assumed that the spatial arrangement of corrole and porphyrin does influence the sensor response. This assumption necessitates to be corroborated by experimental investigation that will be object of further studies.

Results show the great influence of the macrocycle structure and in particular the enhance of sensitivity of free base corrole with respect to the corresponding porphyrin. The increase has been found to correlate with the polar surface area of the volatile compounds, suggesting the improved polar and hydrogen bond interaction in the corrole ring, where in the inner core are present three hydrogen atoms instead of two. As a consequence of the improved polar interactions, the coordination of metal ions for corrole is a less efficient way to improve sensing interaction than porphyrin. On the other hand, iron and manganese complexes always improve the sensitivity of free base porphyrins, but in the case of corrole the improvement is found for manganese and it is less evident for iron. In conclusion, the slight modification of the macrocycle strongly affects the sensing properties, eventually corroles and porphyrins can form an optimal team for sensor arrays, hopefully improving the yet brilliant behavior of porphyrins based artificial olfaction systems.

## References

[1] Editor K., Kadish K., Smith R., Guilard A. (2010). Handbook of Porphyrin Science—Volume 5: Heme Proteins.

[2] Anslyn E. (2007). Supramolecular analytical chemistry. J. Org. Chem..

[3] Stitzell S., Aernecke M., Walt D. (2011). Artificial noses. Annu. Rev. Biomed. Eng..

[4] Di Natale C., Paolesse R., Macagnano A., Mantini A., Mari P., D'Amico A. (2000). Qualitative structure-sensitivity relationship in porphyrins based QMB chemical sensors. Sens. Actuators B.

[5] Di Natale C., Paolesse R., D'Amico A. (2007). Metalloporphyrins based artificial olfactory receptors. Sens. Actuators B.

[6] Aviv I., Gross Z. (2007). Corrole-based applications. Chem. Comm..

[7] Barbe J.M., Canard G., Brandes S., Jerome F., Dubois G., Guilard R. (2007). Selective chemisorption of carbon monoxide by organic-inorganic hybrid materials incorporating cobalt(III) corroles as sensing components. Chem. Eur. J..

[8] Tortora L., Pomarico G., Nardis S., Martinelli E., Catini A., di Natale C., Paolesse R. (2013). Supramolecular sensing mechanism of corrole thin films. Sens. Actuators B.

[9] Wang Y., Akighbe J., Ding Y., Bruckner C., Ley Y. (2012). Meso-tritolylcorrole-functionalized single-walled carbon nanotube donor acceptor nanocomposites for *NO*_2_ detection. Electroanalysis.

[10] Di Natale C., Macagnano A., Martinelli E., Paolesse R., D'Arcangelo G., Roscioni C., Finazzi-Agro A., D'Amico A. (2003). Lung cancer identification by the analysis of breath by means of an array of non-elective gas sensors. Biosens. Bioelectron..

[11] Fuhrhop J.H., Smith K.M., Smith K.M. (1975). Porphyrins and Metalloporphyrins.

[12] Paolesse R., Marini A., Nardis S., Froiio A., Mandoj F., Nurco D., Prodi L., Montalti M., Smith K.M. (2003). Novel routes to substituted 5,10,15-triarylcorroles. J. Porphyr. Phthalocyanines.

[13] Cai S., Licoccia S., D'Ottavi C., Paolesse R., Nardis S., Bulach V., Zimmer B., Shokhireva T.K., Walker F.A. (2002). Chloroiron meso-triphenylcorrolates: Electronic ground state and spin delocalization. Inorg. Chim. Acta.

[14] Steene E., Wondimagegn T., Ghosh A. (2001). Electrochemical and electronic absorption spectroscopicsStudies of substituent effects in iron(IV) and manganese(IV) corroles. Do the compounds feature high-valent metal centers or noni-nnocent corrole ligands? Implications for peroxidase compound I and II intermediates. J. Phys. Chem. B.

[15] Ballantine D., White R., Martin S., Ricco A., Zellers E., Frye G., Wohltien H. (1997). Acoustic Wave Sensors.

[16] D'Amico A., Di Natale C. (2001). A contribution on some basic definitions of sensors properties. IEEE Sens. J..

[17] Ertl P., Rohde B., Selzer P. (2000). Fast calculation of molecular polar surface area as a sum of fragment-based contributions and its application to the prediction of drug transport properties. J. Med. Chem..

[18] Kim D., Kim J., Jung D.H., Lee T.B., Choi S.B., Yoon J.H., Kim J., Choi K., Choi S.-H. (2007). Quantitative structure-uptake relationship of metal-organic frameworks as hydrogen storage material. Catal. Today.

[19] Jolliffe I. (2002). Principal Component Analysis.

